# UCHL1 acts as a potential oncogene and affects sensitivity of common anti-tumor drugs in lung adenocarcinoma

**DOI:** 10.1186/s12957-022-02620-3

**Published:** 2022-05-11

**Authors:** Jianbo Yao, Abdusemer Reyimu, Ao Sun, Zaxi Duoji, Wubi Zhou, Song Liang, Suxia Hu, Xiang Wang, Jingjing Dai, Xiaoguang Xu

**Affiliations:** 1 College of Life Sciences, Anhui Medical University, Hefei, Anhui 230032 People’s Republic of China; 2 Medical College, Anhui University of Science and Technology, Huainan, Anhui 232001 People’s Republic of China; 3 Class 11, Grade 2018, Clinical Medicine, Nanjing Medical University, Nanjing, Jiangsu 223300 People’s Republic of China; 4 Research Center of High Altitude Medicine, Naqu, Tibet, China, People’s Hospital of Naqu Affiliated to Dalian Medical University, Dalian, Tibet 852000 People’s Republic of China; 5 Department of Pathology, The Affiliated Huaian No.1 People’s Hospital of Nanjing Medical University, Huaian, Jiangsu 223300 People’s Republic of China; 6 Department of Medical Laboratory, Second branch, The Affiliated Huaian No. 1 People’s Hospital of Nanjing Medical University, Huaian, Jiangsu 223300 People’s Republic of China; 7 Department of Medical Laboratory, Huainan First People’s Hospital, The First Affiliated Hospital of Anhui University of Science and Technology, Huainan, Anhui 232007 People’s Republic of China; 8 Department of Pediatrics, The Affiliated Huaian No. 1 People’s Hospital of Nanjing Medical University, Huaian, Jiangsu 223300 People’s Republic of China; 9 Department of Hematology, The Affiliated Huaian No. 1 People’s Hospital of Nanjing Medical University, Huaian, Jiangsu 223300 People’s Republic of China

## Abstract

**Background:**

Lung adenocarcinoma is the leading cause of cancer death worldwide. Recently, ubiquitin C-terminal hydrolase L1 (UCHL1) has been demonstrated to be highly expressed in many tumors and plays the role of an oncogene. However, the functional mechanism of UCHL1 is unclear in lung adenocarcinoma progression.

**Methods:**

We analyzed the differential expression of the UCHL1 gene in lung adenocarcinoma and normal lung tissues, and the correlation between the UCHL1 gene and prognosis was also analyzed by the bioinformatics database TCGA. Meanwhile, we detected and analyzed the expression of UCHL1 and Ki-67 protein in a tissue microarray (TMA) containing 150 patients with lung adenocarcinoma by immunohistochemistry (IHC) and clinicopathological characteristics by TCGA database. In vitro experiments, we knocked down the UCHL1 gene of A549 cells and detected the changes in cell migration, invasion, and apoptosis. At the same time, we analyzed the effect of UCHL1 on anti-tumor drug sensitivity of lung adenocarcinoma by a bioinformatics database. In terms of the detection rate of lung adenocarcinoma indicators, we analyzed the impact of UCHL1 combined with common clinical indicators on the detection rate of lung adenocarcinoma through a bioinformatics database.

**Results:**

In this study, the analysis of UCHL1 protein expression in lung adenocarcinoma proved that obviously higher UCHL1 protein level was discovered in lung adenocarcinoma tissues. The expression of UCHL1 was closely related to poor clinical outcomes. Interestingly, a significantly positive correlation between the expression of UCHL1 and Ki-67-indicated UCHL1 was associated with tumor migration and invasion. Through executing loss of function tests, we affirmed that silencing of UCHL1 expression significantly inhibited migration and invasion of lung adenocarcinoma cells in vitro. Furthermore, lung adenocarcinoma cells with silenced UCHL1 showed a higher probability of apoptosis. In terms of the detection rate of lung adenocarcinoma indicators, we discovered UCHL1 could improve the detection rate of clinical lung adenocarcinoma and affect drug sensitivity.

**Conclusion:**

In lung adenocarcinoma, UCHL1 promotes tumor migration, invasion, and metastasis by inhibiting apoptosis and has an important impact on the clinical drug treatment of lung adenocarcinoma. In addition, UCHL1 can improve the detection rate of clinical lung adenocarcinoma. Above all, UCHL1 may be a new marker for the diagnosis of lung adenocarcinoma and provide a new target for the treatment of clinical diseases.

## Introduction

Lung adenocarcinoma is a fatal disease, which happens frequently around the world [1]. Great progress has been made in the treatment of lung adenocarcinoma [2], such as surgicalresection [3], ablation [4], and targeted therapy [5] in the last 20 years. So far, the most effective treatment to cure early lung adenocarcinomas is surgical treatment [6]. Nevertheless, as a result of tumor recurrence and metastasis, the survival rate of lung cancer (22%) in the recent 5 years is not optimistic [7]. Thus, it is necessary to deeply study the molecular mechanism of the occurrence and development of lung adenocarcinoma, find new effective treatment methods, and reduce the recurrence rate of lung adenocarcinoma.

Ubiquitin C-terminal hydrolase-L1 (UCHL1), which is usually expressed in the nerve cells and testicles, is a neuroendocrine cell-specific product with the role of scavenging ubiquitin protein [8]. Although UCHL1 is expressed in normal tissues such as neurons, more and more evidence suggests that UCHL1 is upregulated in some human cancers [9] and plays a critical role in cell proliferation, migration, invasion, and anti-apoptosis [10]. Moreover, neurodegeneration [11], cancer [12], and fibrosis [13] are closely related to the disorder of UCHL1 expression. However, the role of UCHL1 remains unclear in lung adenocarcinoma.

Thus, our study aimed to investigate the expression level of UCHL1 in lung adenocarcinoma and its regulatory effect on drug sensitivity and detection rate of lung adenocarcinoma. The purpose of this study was to investigate the mechanism of UCHL1 in lung adenocarcinoma cells by silencing the UCHL1 gene.

Currently, we have found that UCHL1 is associated with poor prognosis by bioinformatics analysis. Immunohistochemical analysis showed that the expression of UCHL1 was positively correlated with Ki-67 and promoted tumor proliferation. Moreover, we found that UCHL1 can promote the migration and invasion of tumor cells in the functional gene knockout experiment and has an important impact on the clinical drug treatment of lung adenocarcinoma. In addition, UCHL1 can improve the detection rate of clinical lung adenocarcinoma. Our results suggested that UCHL1 may be a target for the diagnosis and treatment of lung adenocarcinoma.

## Materials and methods

### Bioinformatics analysis

In order to explore the expression of UCHL1 in lung adenocarcinoma and normal tissues, the expression of UCHL1 was detected in the UALCAN database (http://ualcan.path.uab.edu/analysis.html). The search criteria are as follows: (1) gene symbol: UCHL1; (2) TCGA dataset: lung adenocarcinoma; and (3) links for analysis: expression. The analysis of UCHL1 gene expression, *P* < 0.05, for the difference was statistically significant. Kaplan-Meier plotter online analysis tool was used to analyze the correlation between UCHL1 and overall survival (OS) in patients with lung adenocarcinoma. The search criteria are as follows: (1) gene symbol: UCHL1; (2) split patients by auto select best cutoff; (3) survival: OS; and (4) histology: adenocarcinoma. To explore the relationship between UCHL1 expression and clinicopathological features in lung adenocarcinoma, we downloaded RNA sequence data and clinical information of 483 patients with complete clinical data from TCGA database. The *c*^2^ test was used to analyze the relationship between UCHL1 expression and clinical parameters of patients with lung adenocarcinoma. Then, the receiver operating characteristic (ROC) curve was used to analyze the accuracy of UCHL1 combined with common clinical indicators (ALK, EGFR, TTF1) in the diagnosis of lung adenocarcinoma. RNAactDrug is a comprehensive database of RNAs related to drug sensitivity obtained from multi-omics data. In this study, rnaactdrug was used to predict anticancer drugs associated with UCHL1.

### Tissue microarrays (TMAs) of lung adenocarcinoma

According to the hematoxylin-eosin (HE) staining results of each lung adenocarcinoma tissue section, 150 cases of cancer tissue and normal lung tissue were determined. The micro tissue sampling equipment was used to conduct micro-sampling on the determined tissues, which were arranged in the carrier tank in turn, and liquid paraffin was slowly injected until the tissues were completely coated. After cooling at 4 °C overnight, the chip wax block was finished. Determine whether the quality meets the requirements through the HE re-staining results of TMAs slices. All research experiments involving patient data were approved by the Ethics Committee (approval number: YX-2021-074-01).

### Immunohistochemical analysis

Immunohistochemistry was used to detect the expression of UCHL1 in lung adenocarcinoma. The slices were placed in an oven at 60 °C for 20 min. After removal, the slices were soaked in xylene solution twice for 10 min and then soaked in 100%, 95%, 90%, and 80% ethanol for 5 min. After cleaning with PBS solution, the slices were incubated in 3% hydrogen peroxide for 20 min and then washed with PBS solution. After drying, the normal goat serum was dripped into the slice surface and incubated at 37 °C for 30 min. The primary antibody (UCHL1/#13179 cell signaling technology, Ki-67/ab15580 abcam) was taken out and added into the wet box at 4 °C overnight. After washing with PBS, the labeled second antibody was added and incubated at 37 °C for 30 min. Then, PBS was washed again and added with a chromogenic agent for routine re-staining, dehydration, and transparency. Finally, neutral gum was used to seal the slices, and the color development intensity was observed under the microscope. The following are the immunohistochemical staining intensity criteria: negative (0–1), weak positive (1–2), medium (2–3), and strong positive (≥ 3).

### Immunocytochemistry

The cells were cultured on the cover glass, washed slowly with phosphate-buffered saline (PBS), and fixed with formaldehyde solution. The cells were then washed with PBS buffer, infiltrated with 0.1% Triton X-100, cultured in a closed solution, and incubated with UCHL1 antibody at 4 °C for 12 h. After washing with PBS, the labeled second antibody was added and incubated at 37 °C for 30 min. Then, PBS was washed again and added with a chromogenic agent for routine restaining, dehydration, and transparency. Finally, neutral gum was used to seal the slices, and the color development intensity was observed under the microscope.

### Cell line cultivation

A549 cells were donated by the medical school of Anhui University of Science and Technology. The DMEM medium was used to culture cells, and 100 U/ml penicillin, 10% fetal bovine serum, and 100 mg/ml streptomycin were added. The humidified incubator was set at 37 °C, and the concentration of carbon dioxide was 5%.

### Construction of UCHL1 gene knockout cells

LDN-57444 (LDN, abcam 141487), UCHL1 inhibitor, was used to inhibit the expression of UCHL1 protein in A549 cells. Normal A549 cells were used as a negative control. 5× 10^6^ cells were cultured in each well of six-well plate using a DMEM medium containing 10% FBS until the fusion degree was about 80%. After the cells were cultured in a serum-free medium overnight, LDN was added to the medium to make the final concentration of the drug 10 mm. After 36 h of drug stimulation, the test of knockout effect was carried out.

### Migration and invasion test

As for the cell migration experiment, 5 × 10^6^ cells were cultured in each well of six-well plate using a DMEM medium containing 10% FBS until the fusion degree was about 80%. After the cells were cultured in a serum-free medium overnight, they were scratched with a 200-ml suction tip, and the results were observed every day. For the cell invasion experiment, there was no serum in the DMEM medium, and 1 × 10^5^ cells were suspended in the medium. Cells were placed in the cavity of the insertion chamber of the matrix gel coated with a diameter of 8 mm, and the DMEM medium containing 10% FBS was used as a chemical attractant to fill the cavity. Forty-eight hours later, the cells invading the lower surface were fixed with an appropriate concentration of methanol and finally stained for microscopic examination.

### Detection of apoptosis by flow cytometry

The materials required for the experiment are as follows: 5 ml and 10 ml graduated pipettes, 5 to 1000 μl adjustable single channel micropipettes with disposable tips, beakers, flasks, cylinders necessary for the preparation of reagents glass-distilled or deionized water, benchtop centrifuge, flow cytometer, and annexin V-FITC apoptosis detection kit. The cells were washed with PBS by gently shaking or pipetting up and down. The cells were resuspended in 200 μl binding buffer (1×); The cell density was 4 × 10^6^/ml. Five microliters annexin V-FITC was added to the 195-μl cell suspension. The cells were mixed and incubated at room temperature for 10 min. The cells were washed in 200 μl binding buffer (1×) and resuspended in 190 μl binding buffer (1×). Then, 10 μl propidium iodide (20 μg/ml) was added. Finally, flow cytometry analysis was carried out.

### Western blot

The cells were extracted and lysed in the RIPA lysis buffer. The target protein was obtained by SDS-polyacrylamide gel electrophoresis (SDS-PAGE), and the protein samples separated by electrophoresis were transferred onto the polyvinylidene fluoride (PVDF) membrane. The PVDF membrane coated with the target protein reacted with the specific antibody (UCHL1/#13179 cell signaling technology, Ki-67/ab15580 abcam) and then incubated with the labeled second antibody (KGAA35/37). The results were observed after adding the reaction substrate.

### Statistical analysis

Graphpad prism 7.0 was used for statistical analysis. Analysis of variance, *t*-test, *c*^2^ test, and Dunnett multiple comparison were used to analyze the data. When *p*-value was less than 0.05, there was dramatic difference.

## Results

### UCHL1 expression correlated with prognosis and poor clinical outcomes in lung adenocarcinoma patients

**Fig. 1:**
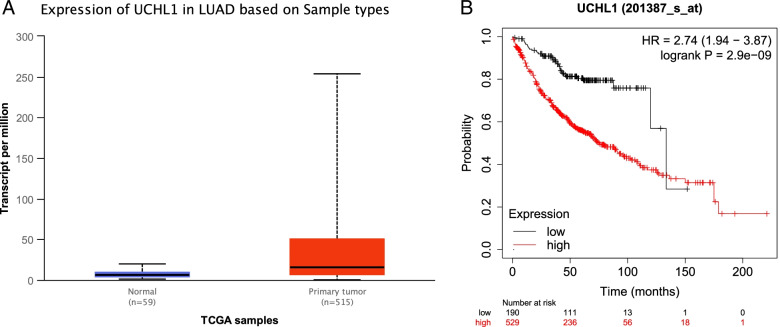
Overexpression of UCHL1 mRNA is associated with survival time in lung adenocarcinoma. **A** UCHL1 mRNA expression in normal and lung adenocarcinoma tissues from TCGA datasets. **B** Kaplan-Meier curve showing overall survival of patients (in percentage) with lung adenocarcinoma, stratified by UCHL1 expression (high- and low-scoring tumors)

**Fig. 2:**
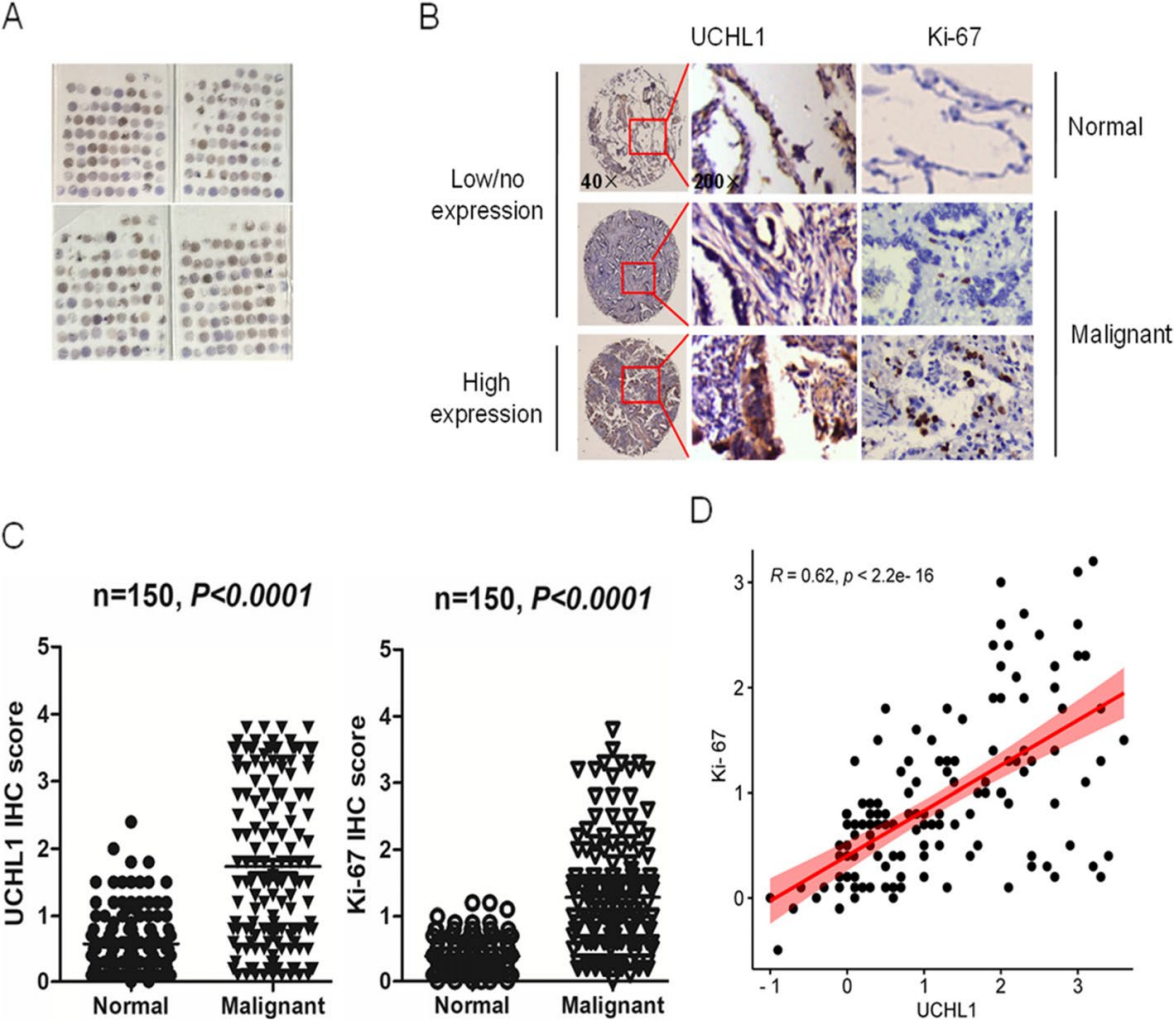
Determination of UCHL1 and Ki-67 in human lung adenocarcinoma tissue microarray. **A** Staining for UCHL1 on lung adenocarcinoma tissue microarrays (TMAs) containing 150 pairs of normal and malignant lung adenocarcinoma tissues. **B** The expression of UCHL1 and Ki-67 in normal and malignant lung adenocarcinoma tissues (magnification × 40, × 200) was recorded. **C** The expression of UCHL1 and Ki-67 was scored according to the percent of UCHL1- and Ki-67-positive cells. The GraphPad Prism7.0 program was performed to compare the relative levels of UCHL1 and Ki-67 between normal and malignant lung adenocarcinoma tissues. Round dots indicate the lung adenocarcinoma sample, and the transverse lines indicate the mean. **D** Correlation analysis between the expression of UCHL1 and Ki-67 in malignant lung adenocarcinoma tissues

To prove the correlation between UCHL1 expression and human disease, through TCGA bioinformatics database analysis, we found that there was a significant difference in the expression of UCHL1 between lung adenocarcinoma and normal lung tissue (*P < 0.05, Fig. 1A, Table 1), and the survival rate of patients with high expression of UCHL1 was significantly lower than that of patients with low expression of UCHL1 (Fig. 1B). Meanwhile, we used a human lung adenocarcinoma TMA for UCHL1 protein expression (Fig. 2A) and revealed that the expression of UCHL1 was lower in the adjacent normal tissues, but higher in the malignant tissues of lung adenocarcinoma patients (Fig. 2B). We aimed to distinguish the differential compartmentalization of UCHL1 expression in tumors with respect to the frequency of UCHL1 expression in cancer cells. The IHC scores indicated that UCHL1 was upregulated in the tumor tissue compared with the normal tissues (Fig. 2B). Further analysis supported that the expression of UCHL1 was positively correlated with Ki-67 (Fig. 2C, D), and Ki-67 was positively correlated with cell proliferation. Moreover, UCHL1 expression was positively correlated with poor clinical survival of patients with lung adenocarcinoma (Fig. 1B). In a word, the above results illustrated that UCHL1 was upregulated and would be a diagnostic indicator for patients with lung adenocarcinoma.

**Table 1:** Correlation between UCHL1 expression and clinicopathological characteristics of 483 patients with lung adenocarcinoma from TCGA database

Factors	Number of cases (*n*)	UCHL1 expression		*c*^2^	*P*
		Low (*n* = 241)	High (*n* = 242)		
Sex				6.785	0.009*
Male	223	97	126		
Female	260	144	116		
Age (years)				1.697	0.193
< 60	131	59	72		
≥ 60	352	182	170		
T				0	1
T1–2	418	209	209		
T3–4	62	31	31		
Tx	3				
N				1.361	0.243
N0	312	163	149		
N1–3	161	75	86		
Nx	10				
M				2.465	0.116
M0	319	154	165		
M1	25	8	17		
Mx	139				
Stage				0.278	0.598
I–II	378	191	187		
III–IV	105	50	55		

Analyses were performed using the *c*^2^ test

*Tx* the primary tumor cannot be determined, *Nx* unable to evaluate, *Mx* unable to evaluate

*P < 0.05

### Knockdown of UCHL1 inhibited lung adenocarcinoma cells migration and invasion

**Fig. 3:**
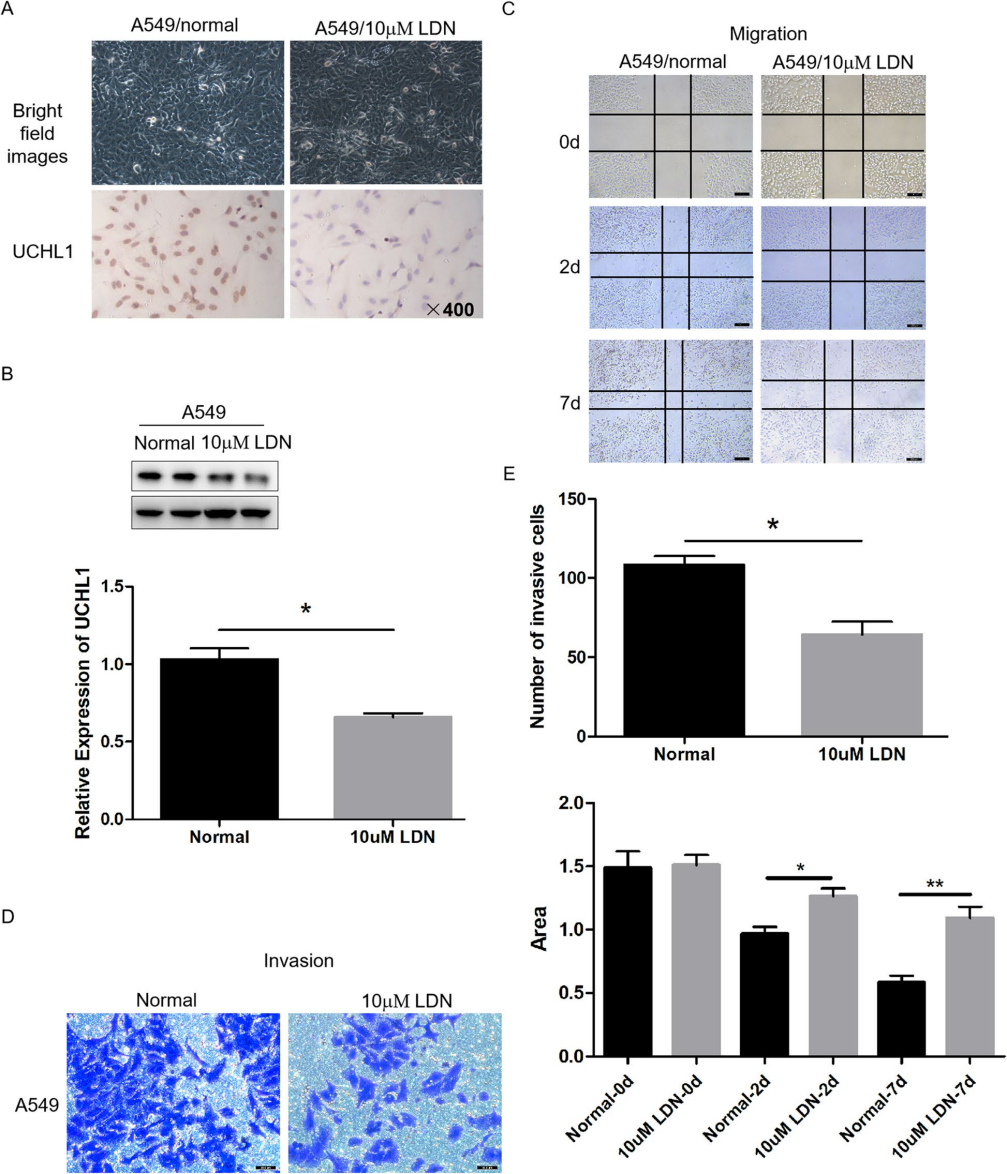
UCHL1 knockdown inhibits cell migration and invasion in vitro. Immunohistochemical analysis (**A**) and western blot analysis (**B**) of interference results of cell lines with 10 mm LDN after 36 h of dosing. The migration (**C**) and invasion (**D**) of cells were detected by scratch and Transwell test (magnification × 400). *P < 0.05, **P < 0.01 compared to the control (**E**)

In order to prove the role of UCHL1 in the metastasis of lung adenocarcinoma, we used the classical scratch and Transwell method to detect the influence of UCHL1 on the migration and invasion of lung adenocarcinoma cells. The results showed that the migration and invasion of lung adenocarcinoma cells were significantly reduced with the silencing of UCHL1 (Fig. 3).

### UCHL1 enhanced cell survival by reducing apoptosis

**Fig. 4:**
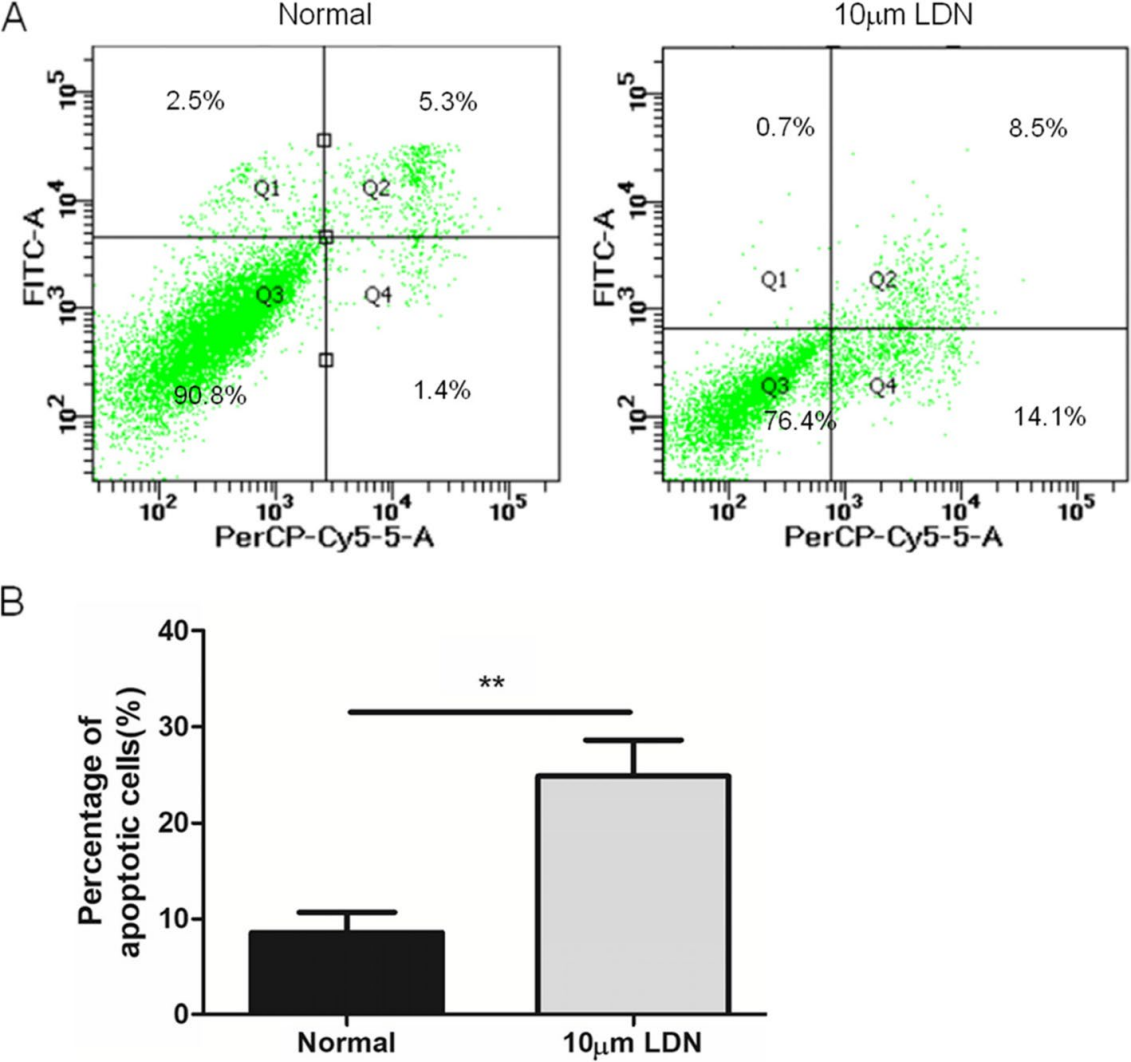
The apoptosis of A549 cells (UCHL1+/−) was detected by flow cytometry in vitro. **A** The apoptosis of normal A549 (UCHL1+) cells and drug-treated A549 (UCHL1−) cells was detected. **B** Statistical analysis of flow cytometry results. (*n* = 3, repeated three times each). **P < 0.01 (Student’s *t*-test)

Flow cytometry showed that normal living cells were not stained with annexin V-FITC and propidium iodide (bottom left). The early apoptotic cells were only stained by annexin V-FITC, but not by propidium iodide (bottom right). The necrotic cells and the late apoptotic cells could be stained with annexin V-FTTC and propidium iodide (top right). The upper left part of the figure shows the detection error within the allowable range. In Fig. 4, when the UCHL1 of lung adenocarcinoma cells was silenced, cell apoptosis was significantly enhanced (**P < 0.01).

### UCHL1 affects the sensitivity of commonly used anti-tumor drugs

RNAactDrug is a comprehensive resource for searching the association between drug sensitivity and RNA molecules at the expression level from the comprehensive analysis of three large drug genome databases (GDSC, CellMiner, and CCLE). Spearman correlation coefficient showed that UCHL1 expression was negatively correlated with perezone, caracemide, bafetinib, and palbociclib and positively correlated with piperlongumine, gefitinib, Embelin, tipifarnib, phenformin, and bosutinib (Table 2). Therefore, perezone, caracemide, bafetinib, palbociclib, and other anti-tumor drugs may become potential therapeutic drugs for lung adenocarcinoma.

**Table 2:** Sensitivity analysis of UCHL1 to commonly used drugs in the treatment of lung cancer

Compound	RNA type	RNA molecule	Omics	Source	Spearman	P*
Perezone	mRNA	UCHL1	Expression	CellMiner	− 0.533803875	0.006029875
Caracemide	mRNA	UCHL1	Expression	CellMiner	− 0.482980911	0.007064843
Bafetinib	mRNA	UCHL1	Expression	CellMiner	− 0.479686548	0.01466201
Palbociclib	mRNA	UCHL1	Expression	CellMiner	− 0.407997669	0.031646287
Piperlongumine	mRNA	UCHL1	Expression	GDSC	0.08744003	0.035600008
Gefitinib	mRNA	UCHL1	Expression	GDSC	0.091381303	0.028871531
Embelin	mRNA	UCHL1	Expression	GDSC	0.097463539	0.047825308
Tipifarnib	mRNA	UCHL1	Expression	GDSC	0.110392871	0.019774916
Phenformin	mRNA	UCHL1	Expression	GDSC	0.110531447	0.001943254
Bosutinib	mRNA	UCHL1	Expression	GDSC	0.115226293	0.003381483

*P < 0.05

### UCHL1 combined with other indicators can significantly improve the clinical diagnosis rate of lung adenocarcinoma

**Fig. 5:**
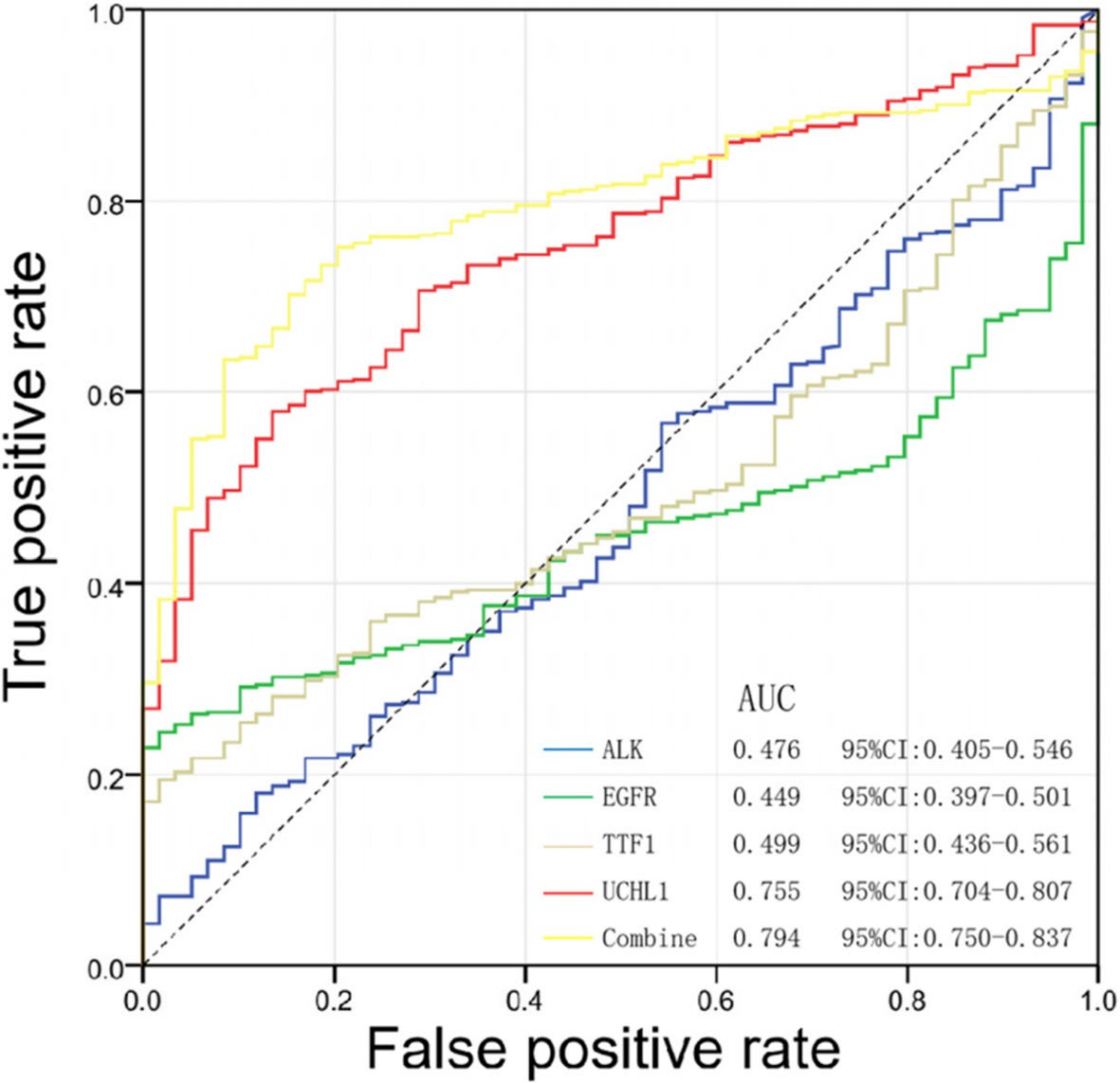
The impact of UCHL1 combined with common clinical indicators on the detection rate of lung adenocarcinoma through a bioinformatics database. AUC, area under the curve

In TCGA transcriptome analysis, the area under the curve (AUC) of ALK, EGFR, TTF1, and UCHL1 were 0.476, 0.449, 0.499, and 0.755, respectively. Compared with ALK, EGFR, and TTF1, UCHL1 has higher predictive accuracy for lung adenocarcinoma. More importantly, the combination of ALK, EGFR, TTF1, and UCHL1 has the highest diagnostic efficiency (Fig. 5).

## Discussion

Lung adenocarcinoma (LUAD) is one of the major subtypes of lung cancer that is associated with poor prognosis [14, 15]. At present, the literature reports that the occurrence and development of lung adenocarcinoma are closely related to circular RNA [16–18], microRNA [19, 20], and N6 methyladenosine [21]. Some scholars have also found some genes related to the differences and prognosis through biological information analysis [15, 22–24], for example, EGFR, KRAS, PIK3CA, STEAP1/2, HOXA11, and TCN1. In addition, some scholars analyze its application value in clinical diagnosis and treatment by constructing a new prognostic model [14, 21, 25, 26]. Therefore, the combination of bioinformatics technology to find possible diagnostic and prognostic markers of lung adenocarcinoma has become the key point.

It has been found that UCHL1 is highly expressed in different kinds of tumor cells [27]. In our study, the expression pattern of UCHL1 protein in normal and lung adenocarcinoma tissues was further interpreted. The results showed that the expression of UCHL1 protein in lung adenocarcinoma was higher than that in normal tissues. In addition, the expression of UCHL1 was positively correlated with Ki-67, and it suggested that UCHL1 was related to cell proliferation. Consistent with previous bioinformatics analysis, silencing of UCHL1 is associated with tumor cell proliferation, migration, invasion, and poor prognosis of patients. The current research is focused on how to interfere with the regulation of UCHL1 in tumor cells. For instance, Parkinson disease uses UCHL1 as a substrate and as a regulator to accelerate the degradation of UCHL1 through the autophagy system [28]. In addition, the expression of UCHL1 is also regulated by lncRNA [29]. The control of UCHL1 translation can be achieved by the insertion of sineb2 repeats by lncRNA [30]. The downregulation of UCHL1 may be mediated by cPKCγ [31]. In addition, LDN pox, a specific inhibitor, can inhibit the activity of UCHL1 cells [32]. Moreover, it has been reported that the decrease of UCHL1 may increase the sensitivity of the interaction between anti-tumor drugs and tumor cells [33]. So far, there is a limited understanding of the mechanism of UCHL1 regulation in lung adenocarcinoma, which needs further study.

UCHL1 has been proved to be able to induce several types of tumors [34]. In particular, UCHL1 promotes TGF-β signal transduction by acting on the TGF-β signaling pathway and Smad2 signaling pathway, thus inducing breast cancer cell metastasis [12]. UCHL1 gene silencing limits the proliferation of endometrial cancer cells and delays the cell cycle [35]. UCHL1 promotes the growth of melanoma cells by activating the activity of protein kinase and activating the signal pathway [36]. In addition, the mechanism of UCHL1 in oncogenesis and development is still unclear. Our results indicate that the proliferation, migration, and invasion of lung adenocarcinoma cells are reduced with the silencing of UCHL1 gene, which further explains the carcinogenic role of UCHL1 in tumors. However, the specific mechanism of UCHL1 in lung adenocarcinoma is still unclear.

The regulatory effects of UCHL1 on autophagy and apoptosis have been reported [37, 38]. It has been reported that UCHL1 plays an important role in the occurrence and development of neurological diseases and other diseases [8]. LDN-induced downregulation of UCHL1 expression and upregulation of autophagy of oligodendrocytes lead to apoptosis [39]. UCHL1 can prevent autophagy (autophagic cell death) and promote cell proliferation by inhibiting apoptosis [40]. Furthermore, the effect of UCHL1 on apoptosis of lung adenocarcinoma cells has been shown for the first time.

In terms of the detection rate of lung adenocarcinoma indicators, we discovered UCHL1 could improve the detection rate of clinical lung adenocarcinoma and affect drug sensitivity. ALK, EGFR, and TTF1 are commonly used to detect lung adenocarcinoma. In order to test the detection efficiency of UCHL1 for lung adenocarcinoma in this study, we analyzed the detection rate of lung adenocarcinoma in a bioinformatics database combined with common clinical indicators. It was found that UCHL1 could distinguish cancer tissue from normal tissue. However, the combined diagnostic efficiency of UCHL1 and common indicators is excellent, suggesting that UCHL1 may have a high clinical application value [41]. Drug sensitivity analysis showed that perezone, caracemide, bafetinib, and palbociclib were negatively correlated with the expression of UCHL1, suggesting that these drugs have potential inhibitory effects on UCHL1. In vitro studies have reported that the natural product of perezone has an antitumor effect [42]. Caracemide was also used in the treatment of advanced renal cell carcinoma earlier [43]. In the past, bafetinib has been used in clinical trials in patients with leukemia and prostate cancer and in the treatment of leukemia [44]. Clinical studies in recent years also show that the use of palbociclib can prolong the overall survival of breast cancer patients [45].

## Conclusion

In lung adenocarcinoma, UCHL1 combined with clinical lung tumor detection indicators improves the detection rate of lung adenocarcinoma, provides sensitive indicators for the early diagnosis of lung adenocarcinoma, and provides possible targets for the development of targeted drugs for lung adenocarcinoma.

## Data Availability

The datasets supporting the conclusion of this article are included within the article.

## Abbreviations

UCHL1: Ubiquitin C-terminal hydrolase L1
TMAs: Tissue microarrays
TCGA: The Cancer Genome Atlas
IHC: Immunohistochemistry
PBS: Phosphate-buffered saline
